# A framework to observe and evaluate the sustainability of human–natural systems in a complex dynamic context

**DOI:** 10.1186/2193-1801-3-618

**Published:** 2014-10-18

**Authors:** Niranji Satanarachchi, Takashi Mino

**Affiliations:** Graduate Program in Sustainability Science-Global Leadership Initiative (GPSS-GLI), Graduate School of Frontier Sciences, The University of Tokyo, Tokyo, Japan; Department of Socio-cultural Environmental Studies, Graduate Program in Sustainability Science-Global Leadership Initiative (GPSS-GLI), Graduate School of Frontier Sciences, The University of Tokyo, Tokyo, Japan

**Keywords:** Sustainability evaluation, Complex dynamics, Sustainability contexts, Sustainability boundaries, Layer view-based method, Dimensional view-based method

## Abstract

This paper aims to explore the prominent implications of the process of observing complex dynamics linked to sustainability in human–natural systems and to propose a framework for sustainability evaluation by introducing the concept of sustainability boundaries. Arguing that both observing and evaluating sustainability should engage awareness of complex dynamics from the outset, we try to embody this idea in the framework by two complementary methods, namely, the layer view- and dimensional view-based methods, which support the understanding of a reflexive and iterative sustainability process. The framework enables the observation of complex dynamic sustainability contexts, which we call observation metastructures, and enable us to map the contexts to sustainability boundaries.

## Introduction

Sustainability is an evolving concept in an age of complexity. Human–natural systems where unsustainability issues were observed are highly complex and dynamic (Kates et al. 2001; Holling 2004; Swart et al. 2004; Komiyama and Takeuchi 2006; Ostrom 2007; Morin 2008; Reid et al. 2010). Sustainability or unsustainability conditions in these systems are diverse and change across space and time. These diversities and changes are not readily visible, which makes observing and evaluating sustainability in them a challenging task.

Sustainability also has significant conceptual diversity (Neumayer 2003; Bell and Morse 2008; Espinosa et al. 2008; Jerneck et al. 2011).^a^ Incorporating both holistic and context-specific conceptual understanding is necessary for rigorous interpretations of sustainability (Meppem and Gill 1998; Kates et al. 2001; Clark and Dickson 2003; Mihelcic et al. 2003; Swart et al. 2004; Espinosa et al. 2008). Failing to do so often results in specific but not generally representative interpretations, or overly simplified or generalized interpretations.

These challenges are also visible in frameworks and methodologies used to observe and evaluate sustainability. In general, these frameworks and methodologies focus on interpretations of the static state of a system. They also often support the analysis of parts and the specific processes that can scrutinize individual aspects of complexities. Conversely, they can also produce generalized overviews that aim to reduce the complexities. However, the conceptual nature of complex dynamics demands that the frameworks and methodologies should adequately internalize both of these ends.^b^ This means that in the face of complex dynamics, observation processes play a key role in the evaluation of sustainability.

The observation of multiple sustainability or unsustainability contexts is a significant step in internalizing complex dynamics linked to sustainability in an evaluation process. Observing the contexts involves awareness of diverse sustainability principles, and in addition, awareness of sustainability or unsustainability conditions in systems across space, time, and organizing relationships.^c,d^

Taking these factors into account, our first aim is to explore the prominent implications of the process of observing complex dynamics linked to sustainability in human–natural systems by focusing on some of the key features of complexity, complex systems, and complex dynamic patterns and mechanisms. We then propose an evaluation framework that focuses on observing multiple complex dynamic sustainability contexts by introducing the concept of ‘sustainability boundaries’. Arguing that both observing and evaluating sustainability involves a thorough awareness of complex dynamics from the outset, we incorporate this idea into the framework by using two observational methods, namely, the layer view- and dimensional view-based methods. These methods help to map contextual sustainability understanding to sustainability boundaries.

## Observing sustainability boundaries in complex dynamic contexts: a framework approach

### Observing complex dynamic contexts

Considering the nature of the planet Earth and human dependency on natural resources, the systems relevant for sustainability evaluation can be identified as human–natural systems. These systems are connected in space and time through system–subsystem relationships that include complex dynamic organizing relationships. One of the key documents in sustainability science, *Sustainability Science: The Emerging Research Program* (Clark and Dickson 2003), emphasizes the importance of harnessing the dynamic interactions with attention to how social changes shape the environment and how environmental changes shape society. Earlier, Costanza and Patten (1995) suggested that much of the sustainability discussion at the time was misdirected because researchers failed to account for the range of interrelated temporal and spatial scales over which the concept must apply. They further stated that a sustainable system is one that persists, which led to the questions by Howe (1997): what systems, subsystems, or characteristics persist?, how long must a system persist to be considered sustainable? highlighting that any subsystem is not indefinitely sustainable because this would eliminate evolutional adaptations. These observations indicate that, in addition to system–subsystem relationships, it is necessary to give in-depth attention to other forms of complex dynamic relationships in sustainability interpretations.

Complex dynamics have a wide spectrum of meaning (Morin 2008; Wells 2012). The closely related concept of a ‘complex system’ has its conceptual origin in the complexity theory of Leibniz and von Bertalanffy (Cillers 1998, 2002; Taylor 2002; Morin 2008; Wells 2012). This was later developed and adapted in numerous fields such as natural science, ecology (Capra 1997; Gunderson 2001; Berkes et al. 2003), social science (Bailey 1994; Miller and Page 2009), and philosophy (Bateson 1979; Morin 1992, 2008). In recent times, the idea of complexity has had widespread popularity outside the sciences, requiring researchers to be careful of the dangers of clarification, simplification, and overall rapid reduction of knowledge (Morin 2008). Here, it is useful to distinguish between the notions of ‘complex’ and ‘complicated.’ If a system—despite the fact that it may comprise a huge number of components—can be completely described in terms of its individual constituents, then it is merely *complicated.* However, in a complex system, the interaction of constituents of the system, and the interaction between the system and its environment are of such a nature that the system as a whole cannot be fully understood simply by analyzing its components. This is also the case with the systems that we observe in the evaluation of sustainability. Additionally, complex systems are inherently dynamic in nature. One reason is that these are open systems; they interact with their environment, in terms of not only matter and energy, but also information. These systems adapt to changes in the environment; therefore, their internal structure is influenced by external conditions that make a clear distinction between the ‘inside’ and ‘outside’ of the system problematic. Moreover, the system relationships are not fixed, but can change often as a result of feedback-based self-regulation and self-organization, where such processes can result in novel *emergent properties* of the system (Cillers 1998; Miller and Page 2009; Morin 2008). In this light, the human–natural systems’ patterns of interactions are evolutional and appear as complex dynamic paths over time. Such paths are visible not only because of the complex dynamic nature of the systems, but also because of the complex dynamics tied to the process of observing them as systems.

Concerning the observation of systems, Mebratu (1998) indicated that an epistemological flow runs across different versions of sustainability because the relationship between the part and the whole in systems concerning sustainability is often not properly captured. A well-elaborated simile (a tapestry) that describes the process of observation with regards to parts and wholes can be found in Morin (2008), who describes three stages of complexity that influence observation as follows:“

(i) *In the first stage of complexity*, we have simple knowledge that does not explain the properties of the whole. A banal observation that has consequences is not banal; the tapestry is more than the sum of its threads. The whole is more than the sum of its parts.
(ii) *In the second stage of complexity*, the fact that there is a tapestry means that the qualities of this or that type of thread cannot be expressed fully. The threads are inhibited or virtualized. Therefore, the whole is less than the sum of its parts.
(iii) *In the third stage of complexity*, problems are posed relating to our capacity to understand and the structure of our thoughts. The whole is simultaneously more and less than the sum of its parts.

In this tapestry as in an organization, the threads are not placed randomly, they are organized to make a canvas; i.e., they have a synthetic unity where each part works together with the whole. The tapestry itself is a perceptible and knowable phenomenon that cannot be explained by any simple law” (Morin 2008, p.60).

Similarly, observing sustainability involves a cognitive process of organizing the knowledge related to human–natural systems’ evolutionary paths, general sustainability principles, system-specific sustainability or unsustainability conditions, and to complexities involved in the observation process. In the field of sustainability, there have been milestone works to address the complex dynamics of human–natural systems along with their implications of sustainability in those systems^e^ (López-Ridaura et al. 2002; Ostrom 2007; Liu et al. 2007). In addition, methodologies such as transdisciplinary research (Scholz and Tietje 2002) and soft systems methodology (Wilson 2001) have focused on necessary dialogue among stakeholders in exposing the complexities in the decision-making process. In actual practice, however, sensitivity to the process of observation of complex dynamics seems to still be lacking, which subsequently adds up to erroneous and incomplete interpretations and evaluations of sustainability. Therefore, as a way of translating complex dynamics linked to sustainability understandings to a sustainability-evaluation process, we propose a framework that embeds a methodology to observe and evaluate complex dynamic sustainability contexts by using a concept of ‘sustainability boundaries’.

### Sustainability boundaries

The term ‘boundary’ is usually used to demark something from what it is not. In relation to sustainability understanding, a sustainability boundary would mark what is sustainable and what is not. Interpreting sustainability in relation to boundaries is not new. As a concept, sustainability often addresses location-specific *facets of sustainability*, such as development, growth, technological efficiency, environmental and cultural conservation, ensuring socionatural resilience, and so on, that often represent prominent sustainability issues, their solution trajectories, or positive/negative characteristics of systems that lead towards sustainable/unsustainable conditions.

The discourse of sustainable development and sustainability has been enriched with conceptual interpretations that focused more on *explicit boundaries*. Traditionally, the very idea of a sustainability boundary is directly related to limitations. Early dialogues on the dependency of human functions on natural resources addressed explicitly the *physical limitations* on Earth. They brought out terminologies such as *limits to growth*, which signifies an upper cap on stresses on global physical resources (Meadows et al. 1972). The term ‘*our common future*’ implied a future limited space in which humanity operates within these predominantly physical limitations. Additionally, the concept of the *ecological footprint* (Wackernagel 1996) has given a strong metaphorical representation and a quantitative basis to many of these physical limitations. Recently, stemming from ever-increasing global catastrophes, *planetary boundaries* have highlighted the significance of being aware of physical thresholds, and also of the complex dynamics that can trigger rapid movements towards these thresholds (Rockström et al. 2009). The concept of planetary boundaries also implicitly suggests *a safe boundary for human actions* that highlights the connectivity of human systems to the wider ecological setting very well. Such limitations do not remain in physical forms alone, but rather extend to biological forms—especially to human-related forms. These include not only the readily visible boundaries of technologies and institutions, but also those of knowledge, views,^f^ capacities, wisdom, and aesthetic sensitivity that have multiple trajectories. Unlike physical limitations, which mark clear thresholds (for instance in the case of viewing the world as one planetary system), the other limitations tend to be those that can be overcome or be reached a stage where they are no longer regarded as limitations (e.g., new knowledge, different levels of understanding, and capability [Sen 2009]). Additionally, some are easily recognizable and communicable while others, like wisdom and views, tend to be subtler and may not be easily recognizable as limitations. Even further, our very understanding of concepts such as aesthetic sensitivity (Bateson 1979, 2000; Kagan 2010, 2011) and their roles in forming perceptions of system interlinks is quite limited. Nonetheless, it is possible to recognize that there are both hard and soft types of boundaries connected to different sustainability contexts. Further, the notion of sustainability implicitly carries long-term perspectives and the need for avoidance of future catastrophic conditions across time, which strongly implies that sustainability contexts need to be visualized with sensitivity to their continuous nature across time. For these reasons, we hold that boundaries that represent context-based sustainability or unsustainability understanding need to be visualized as complex and evolving entities.

### Methods for observing sustainability boundaries

To observe these complex and evolving sustainability boundaries, we incorporate two complementary observation methods, namely, the layer view- and dimensional view-based methods. Both methods enable the observer to recognize the complex dynamics of systems and the process of observation. The layer view-based method enables us to observe sustainability contexts reflexively by focusing on system relationships in a relatively fixed time frame. The dimensional view-based method integrates the variable sustainability conditions across time by referring to a set of sustainability principles. The methods are described in detail as follows.

#### Layer view-based method

In the evaluation of sustainability, we often focus on one system of a wider human–natural system, such as an economy, society, or nature, and interpret its sustainability. However, to make rigorous interpretations, it is also necessary to not only focus on that particular system alone, but also to refer to its ‘background’. One group of entities that forms the ‘background’ for a particular focused system is the relationships that it has with its subsystems. Some of those relationships would have significant implications in interpreting its sustainability. Another group is the explicit unsustainability issues observed related to the focused system. Depending on immediacy, proximity, and significance, the ‘backgrounds’ with which a focused system is contextualized varies. As a result, the observed sustainability or unsustainability conditions would change.

**Figure 1:**
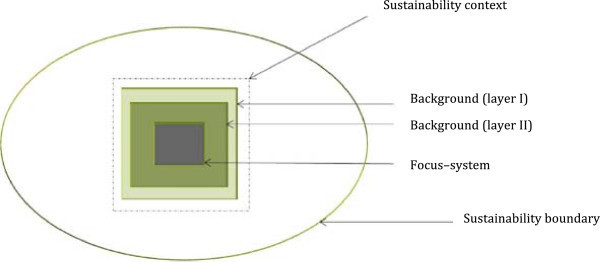
**Visualizing sustainability boundaries with a focus–** **system and the background.** Note: By separating the focus–system from the ‘background’ and observing them together, the focus–system is placed in a bigger context. In addition, by interchanging the focus–system and its ‘background’, the significance of their parts and their relationships to the whole can be captured.

Using the layer view-based method, we aim to strengthen the sustainability observation process by highlighting such variations. To observe the complex dynamic sustainability contexts of a human–natural system, first we propose to differentiate ‘focus–system’ from its ‘background’. An observation process that focuses on one system, by allowing information from other systems to form a background for interpretations about the system, engages separate cognitive distances.^g^ In other words, the ‘background’ functions as a set of layers to provide knowledge about the focus–system (Figure 1). Two forms of knowledge can be obtained about the ‘focus–system’ and its ‘background,’ namely, primary understanding, which represents focused understanding, and subsidiary understanding, which, when connected with primary understanding, can lead to holistic understanding.^h^ The ‘background’ can be seen as storing information to support the primary understanding through subsidiary understanding. By interchanging ‘focus–system’ and ‘background’, and interchanging different ‘backgrounds’ for a particular ‘focus–system’, a holistic sustainability understanding can be achieved.

If compared with the relationship between the parts and the whole in complexity, the process of observation with our proposed differentiation and integration allows an understanding that represents a ‘holistic sustainability understanding’, by focusing first on ‘parts’, and second on ‘wholes’. In this case, systems and the background layers denote ‘parts’, and the emergent understanding gained through the unit of ‘system and background’ denotes the ‘whole’. Furthermore, this process of observation could be seen as a reflexive observation process. Therefore, the observation process supported by the layer view-based method appears as a complex dynamic process in itself.

By this first method of observation, we hope to lay the foundation for obtaining sustainability boundaries for a human–natural system.

#### Dimensional view-based method

##### Overview

Differentiating ‘focus–system’ from its ‘background’ alone, however, is insufficient for the interpretation of the complex dynamic sustainability contexts of a human–natural system. It is also necessary to refer to the sustainability or unsustainability conditions of the unit of ‘system and background’ explicitly. Such conditions can be recognized by observing systems through a set of general sustainability principles^i^ and context-specific sustainability or unsustainability understandings. Further to capture the complex dynamics of systems fully, it is important to observe the variability of contexts, not only across space, but also across time and organizing relationships. Thus, as a complementary and completing step, the framework uses a second method of observation, namely, a dimensional view-based method.

In the past, dimensional approaches have been proposed to frame complex dynamic systems relevant to sustainability that includes the well-known three pillars or dimensions of sustainability (Hawkes 2001; Hopwood et al. 2005; Komiyama and Takeuchi 2006; Gibson et al. 2000). In our case, rather than aiming to define what is sustainable and unsustainable in an all-inclusive manner, as was the purpose of some of these previous instances, we use the dimensional approach to engage the observer in the process of evaluation of sustainability or unsustainability. For this evaluation, the dimensions to be introduced should have the capacity for indicating sustainability or unsustainability, the ability to link general principles and context-specific understanding, the ability to visualize complex dynamic changes, and also the flexibility to be adopted in diverse contexts. In addition, to see the changes readily, the capacity of these dimensions to make these changes visible needs to be considered. Accordingly, we have selected six significant dimensions that can be used to observe sustainability.

##### Description of dimensions

(i) *Sustainability-linked knowledge*
  By sustainability-linked knowledge, we mean knowledge that is predominantly connected to unsustainable issues, and to systems that are experiencing those issues. In a pragmatic sense, the knowledge about resources, well-being, policies, regulations, and artifacts, etc., inform sustainability or unsustainability conditions. In a theoretical sense, there are different types and categorizations found within the knowledge relevant to sustainability, such as philosophy-oriented knowledge as personal, procedural, and propositional knowledge (Polanyi 1974); reality-based knowledge as explicit and tacit knowledge (Polanyi 1966); context-based embedded knowledge as local and disciplinary knowledge (Ramakrishnan 2000; Berkes et al. 2003).^j^ These types inform the varying principles with which a system is interpreted as having sustainability or unsustainability. Collectively, these different knowledge types can be argued as giving variable interpretation grounds with verifiable capacity to the observer. Their significance in the evaluation of sustainability would vary depending on the adopted intervention or research approach, such as problem- or planning-driven interventions and descriptive, analytical, or transformative research methods (Watzlawick 1974; Holzner and Marx 1979; Heylighen 1988; Salas-Zapata et al. 2012; Wiek et al. 2012a, 2012b). Additionally, recognizing changes to knowledge also is significant for the observation of change in sustainability. Gross (2007) distinguishes five different types of dynamics forming knowledge, namely ignorance, nonknowledge, negative knowledge, extended knowledge, and nesciences. Ignorance denotes knowledge about the limits of knowledge in a certain area that can increase with every state of new knowledge. Nonknowledge denotes what is not yet known, but is being considered for future planning. Negative knowledge addresses what is not known, but considered as unimportant or even dangerous. Nescience stands for the lack of any knowledge, which leads to surprises. Extending Gross’s interpretation, we recognize that these different types of knowledge represent interconnected stages that could lead from one to other over time, and by doing so, would change the interpretation of sustainability or unsustainability about a system, hence sustainability boundaries.
(ii) *Sustainability-linked worldview*
  Similar to knowledge, worldviews are closely tied to the understanding process. Worldviews in general and in a conceptual sense can be regarded as a set of images and assumptions that the human system holds in observing reality. Depending on the context, worldviews are associated with a variety of concepts, such as gestalts, mindsets, mental–models, mental–structures/frameworks, and frames of mind (Gardner 1983; Covey 1991; McEwen and Schmidt 2007; Gidley 2010), and often are visible through metaphors, paradigms, inquiries, disciplines, and so on. Koltko-Rivera (2004) describes worldviews as being coherent systems of beliefs that shape how individuals interpret and interact with the world by shaping how they think and, consequently, what they think about it. Worldviews define what can be known and done, and what goals should be pursued, functioning at a level more abstract than the level of theory and observation (Grunig and White 1992). In other words, the agent’s worldview represents its value orientations.^k^ Worldviews form and strengthen metastructures^l^—with which agents observe and analyze their surroundings—by using subtle meaning-making processes (Polanyi and Prosch 1977) and ethical justifications (Heylighen 1988; Funtowicz and Ravetz 1993; Allenby 2006; Armand 2012; Beckers 2012) that are crucial in interpreting sustainability. Further, Van Egmond and De Vries (2011) suggest that sustainable development in the Brundtland definition implies the continuation of certain capabilities, where capabilities among other factors depend on a person’s ‘*value orientation*’ for his or her individual perception of the good life, which means that the idea of sustainability should be grounded upon multiple normative standpoints that the human system holds for its notion of well-being (Van Egmond and De Vries 2011). Also, these worldviews change and develop over time (Lynam 2012), which influences the change in an agent’s sustainability interpretations. Therefore, a worldview can be regarded as operating on a subtle level to define and change the sustainability conditions as well as the sustainability understanding of a system.
(iii) *Resource-related limitation and availability*
  The discourse of sustainability has traditionally been heavily linked with increased attention towards resource limitation (Meadows et al. 1972; Wackernagel 1996). The meaning of resource limitation spans a wide scope to include limitations related to not only the often-discussed hard physical resources, but also other soft forms such as human-related knowledge and technologies. The significance of these resource limitations in characterizing a system’s sustainability varies from one system to another. Generally, limitations could easily create instability in systems and trigger changes in the systems to confront these limitations. These changes could be in the form of short-term adaptations as well as system reorganizations with significant long-term implications. Therefore, limitations not only would define sustainability conditions in a system, but also may trigger significant sustainability or unsustainability changes that alter its path in the long run.
(iv) *Well-being views*
  As the flipside of limitations, general views of well-being specify what conditions individuals and societies consider sustainable or unsustainable (Dasgupta 2001; Neumayer 2004); therefore, well-being views become essential considerations to interpret the sustainability of a human–natural system. The ideas of well-being are old as human discourse, and historically have been reflected in numerous general discussions of the ‘good life’ and ‘good societies.’ Over the years, a multitude of theories that categorize well-being have emerged, for instance the categorization by Dodds (1997) of (a) well-being as a state of mind, (b) well-being as a state of world, (c) well-being as human capacity, and (d) well-being as the satisfaction of underlying needs. There are other similar attempts (Alkire 2002). The implications derived from such a wide scope of interpretations are important not only to achieve a rigorous conceptual basis for sustainability, but also to achieve more-stringent evaluation practices. The limitations of well-being could be understood as directly indicating the sustainability or unsustainability conditions of a system. However, Neumayer (2004) observes that unfortunately, in early conceptual developments, most indicators of well-being have ignored sustainability, and most indicators of sustainability have ignored well-being. Furthermore, it is noteworthy that the gap between the present well-being and the past and future anticipated well-being in general could drive a system’s sustainability or unsustainability changes.
(v) *Policies, rules, regulations, and governing practices*
  Sustainable governance lies at the very heart of the concept of sustainability. The earliest conceptual developments have embedded the responsibility of humans to regulate within limits into sustainability understanding; therefore, they have repeatedly highlighted the need for better governance. In addition to explicit discussions on sustainable governance (Adger and Jordan 2009; Jäger 2009), there are also other significant branches such as global governance (Lövbrand et al. 2009), governing commons (Ostrom 1990, 2007, 2010), adaptive governance (Folke et al. 2005), and reflexive and path-dependent governance (Voß and Kemp 2006; Leach et al. 2010). These address different means of conduct within identified limitations highlighting varieties of formal laws, and social, economic, and political practices with varieties of frameworks. Especially in a dynamic context where past, present, and future are important considerations, rules and regulation support concrete envisions of possible future solution spaces (Wiek and Binder 2005) at decision points. Depending on the existing policy and governing structures, these solution spaces would be envisioned differently in different contexts and mobilize different sustainability or unsustainability paths.
(vi) *New creations, innovations, and artifacts*
  In general, new creations, innovations, and artifacts have the capacity to shape human interactions and determine the paths in which societies move. They play a prominent role in a human system’s capacity to create, co-create, and transform itself. A society’s orientation with respect to this dimension also shapes its anticipation of future possibilities; therefore, this dimension influences how both present and future sustainability boundaries are perceived. Also it is well recognized that we live in the Anthropocene (Crutzen 2006; Lövbrand et al. 2009; Rockström et al. 2009), which means that creations and artifacts become increasingly distinctive in directing the thinking and behavior patterns of agents, and as a result, directing the human–natural systems’ sustainability changes.

##### Prominent characteristics of dimensions, and their role in the understanding and evaluation of sustainability

Based on their role in forming and changing sustainability boundaries, these dimensions can be placed into three groups. We recognize that sustainability-linked knowledge and sustainability-linked worldviews form the underlying understanding process; therefore, they are the foundational dimensions in an evaluation process. In general, sustainability gains its meaning from human interpretations of a system. These interpretations are influenced by both personal knowledge and worldviews. For instance, new knowledge about an issue interacts with one’s internal views on that issue to form new interpretations. These preliminary interpretations are important in the way that they provide reference frames—in other words, metastructures for further observations. Therefore, to a certain extent, they provide *temporary stable grounds*. We use the term ‘temporary stable grounds’ because, upon making interpretations, agents form relatively stable views of sustainability^m^ even while the ideas themselves change and transform. In our view, the process of transforming these temporary stable grounds demands complex thinking.^n,o^ On the other hand, resource-related limitations and well-being views form the basis of sustainability. They are closely connected to its fundamental definition. The agent’s views on resource limitations and well-being often determine what are considered to be prominent unsustainability issues, and what are considered as positive attributes for the integrity of a system. They are easily visible and act as entry points for the observation of sustainability or unsustainability. Further, as observed by Adger and Jordan (2009), these dimensions also could give contradictory and paradoxical interpretations to sustainability. Once the sustainability or unsustainability conditions are interpreted, the operational practices that can be adopted by a human system are represented by the final two dimensions. The policies, laws, regulation and governing practices enable human systems to manage within limitations. New creations, innovations and artifacts enable them to go beyond already recognized limitations. Likewise, the proposed dimensions would not be alike in their role in the process of evaluating sustainability.

At this point, it is also important to note that none of these dimensions could be discussed without observing their embeddedness in the human–natural systems, and special complex dynamic features linked to this embeddedness. Furthermore, apart from the dimensions that are described here, there may be other prominent dimensions that could represent general principles of sustainability, or that could represent specific contexts. Therefore, the role of dimensions in influencing the understanding and subsequently the evaluation process is very much context-bound; however, identifying some of their general characteristics is helpful to explore their functions further.

(i) *The dimensions aggregate varying conditions that indicate the sustainability or unsustainability of a system.* There are varying types of knowledge that have strong implications on informing what is sustainable and what is not. Multiple and sometimes conflicting data and information are available around a specific issue. Also, there are different worldviews linked to sustainability or unsustainability such as materialistic and minimalistic views, and well-being views also change from person to person, across societies, and across time spans. Varying types of resources such as natural, human, and man-made resources give a wide scope of interpretation for the availability of resources. In the same manner, different governing practices, rules, and regulations exist. For instance, economic practices include local economies as well as global market economies and resource-governing rules include local soft rules and formal state rules. In terms of new innovating pathways, there also exist multiple possibilities that can tilt a system towards and away from sustainability. These entities could be considered as different points of observation along the dimensions. While it may not always be feasible to give measurable units, these units could be used to recognize either quantitative values or qualitative interpretations that indicate specific sustainability or unsustainability conditions. These conditions would be heavily context-bound.
(ii) *Observations of systems with respect to dimensions in a fixed time frame can lead to different interpretations of sustainability within that time frame.* Such interpretations can refer to varying points along the dimension, and to varying focus–system and background relationship combinations. The dimensions provide windows of observation for the later.
(iii) *Observations made of systems with respect to different dimensions along time, allows the recognition of systems’ time-dependent complex dynamic changes.* For example, changes in values/attributes can represent system changes that mark significant emergent changes that lead to new sustainability or unsustainability states, or the ones that solidify the current state. In-depth attention to these patterns and mechanisms allows us to see which dimension or dimension combination is likely to trigger a significant change in the system.
(iv) *Variability along dimensions or observations of variability generated through their interactive influences is important in recognizing sustainability changes in systems.* For instance, change in knowledge over time can verify the wrong use of a resource; however, without interventions such as policy and regulation change, the knowledge alone would not lead to change in initial practices. Such policy or regulation changes would also rely upon active changes in other dimensions. Therefore, these changes would involve time lags and interconnected feedback processes, which means that changes related to one dimension would not necessarily lead to immediate changes in other dimensions. Additionally, it is noteworthy that changes observed in systems with respect to each dimension are characteristic to it; therefore, the time lags involved are different from each other. The usual bird’s eye view that we employ to scrutinize systems tends to miss changes across time in particular. This makes changes between the system’s sustainability states appear to be a result of change in sustainability or unsustainability conditions with similar speeds, patterns, and mechanisms. However, the feedback loops that work along and across dimensions may trigger different dynamic patterns in system.
(v) *Dimensions may have the capacity to drive the changes in a system.* While providing different contexts to observe the sustainability of the system, depending on the context, some of these dimensions also may have the capacity to make significant sustainability or unsustainability changes by acting as driving forces. The implication is that right selection of dimensions to observe systems would enable us to recognize not only significant sustainability or unsustainability conditions, but also the factors that can drive significant changes in the system.

## Synthesis of the two observation methods in the framework and further discussion

**Figure 2:**
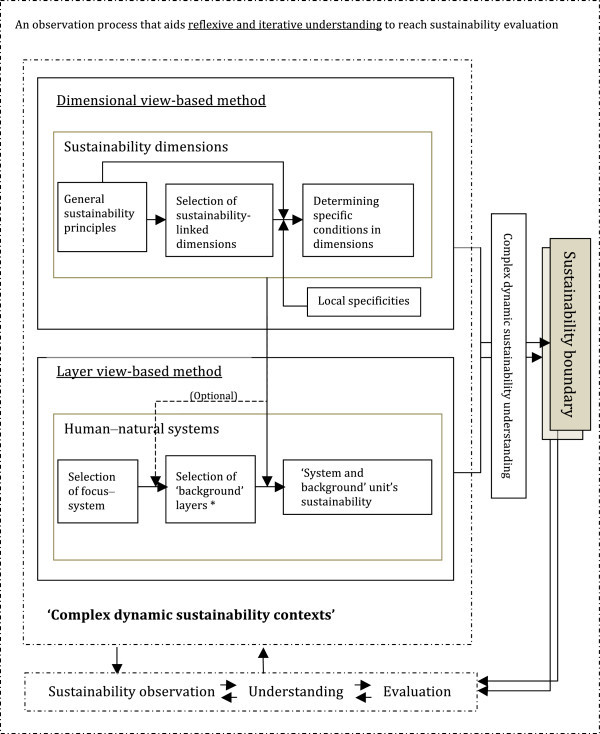
**Detailed illustration of the observation process supported by the framework.** Note: *The ‘background’ layers are selected by referring to system relationships and unsustainability issues.

**Figure 3:**
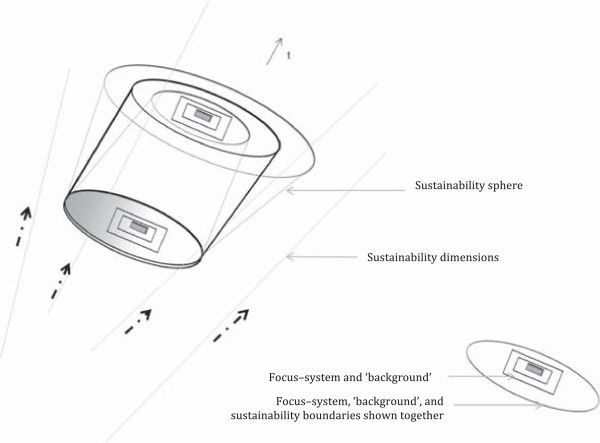
**Visual illustration of the conceptual framework.** Note 1: The proposed framework maps sustainability contexts to conceptual sustainability boundaries. Apart from acting as windows of observation for sustainability boundaries, the dimensions also represent change mechanisms such as driving forces between consecutive states (shown by dashed arrows in the diagram). Some of these change mechanisms would lead to the co-creation of new sustainability states for the system. Such changing patterns in sustainability boundaries across time can be visualized as a spiral, which we refer to as a sustainability sphere. Note 2: Only four dimensions are shown to maintain the clarity of the picture. As illustrated, the changes triggered by the dimensions can hypothetically expand or contract the sustainability sphere. The figure shows three scenarios; i.e., contracting, constant, and expanding spheres over time.

Once the layer view- and dimensional view-based methods are combined, an overall framework can be proposed for the observation of sustainability contexts (Figures 2 and 3). By addressing different ‘system and background’ units by referring to different system relationships^p^ and different dimensions along the time line, multiple sustainability contexts can be reached. A sustainability context in this case resembles a metastructure of observation. Together, these layers and dimensions generate a metastructure with which a focus–system can be evaluated. Two types of metastructures are supported: one describes a system’s sustainability and sustainability changes; the other describes the changes to an observer’s understanding process. Using a set of metastructures or contexts, the framework actively engages the observer by making him or her become aware of the observation process. Such an awareness could lead to positive outcomes, including making the observer’s assumptions in their evaluation more visible. In addition, by referring to a context, the observer is localizing their general understanding of sustainability to gain specific interpretations that would in turn lead to a holistic understanding. According to Polanyi and Prosch (1977), localizing related to understanding is affected by the available information, awareness, and other similar factors. Such a localizing process can be different for each agent with their specific knowledge, expertise, preunderstanding, mental frames, or future orientation. By adopting the framework, such diverse localizing processes could be made visible.

The proposed framework maps sustainability contexts to conceptual sustainability boundaries. Furthermore, the changes in the boundaries are made visible as changes between relatively stable levels and changes within such levels. The idea of emergence seems to suggest that the process of change can occur in steps and can create strong outcomes as new temporal stable states. With relation to sustainability, these temporal stable states could represent new epochs or levels of realities^q^ that describe the sustainability of a system—that is, the new sustainability states of a system. It could also create weaker outcomes such as new sustainability or unsustainability conditions within the same state. Further it could create causal ‘laws’ that function as driving forces across states. Some of these driving forces would have the capacity to degenerate old sustainability states and co-create new sustainability states for that particular system. With such patterns of change, obtaining boundaries along time can be visualized as spiraling boundaries, which we refer to as a sustainability sphere. The space within the sphere represents a sustainable operating space, and, by interchanging layers and interchanging dimensions, hypothetically, the space can be visualized as expanding or contracting over time (Figure 3).

Overall, there are several significant roles that the framework plays in a complex dynamics-focused sustainability evaluation process. Of these roles, the prominent ones are as follows:

**Figure 4:**
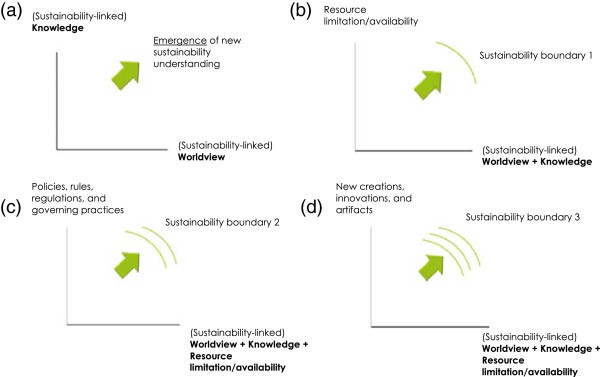
**Illustration of the interplay of dimensions to form an iterative understanding.** Note: **(a)** Sustainability understanding is mainly formed by the interplay of the two dimensions of sustainability-linked knowledge and sustainability-linked worldview; **(b)**, **(c)**, **(d)** Examples of how to reach consecutive sustainability boundaries by referring to different dimensions. In each instance, the previous understanding continues to inform the new understanding.

(i) *Helps to recognize multiple sustainability contexts and multiple sustainability boundaries.* One of the fundamental roles of both layers and dimensions in this framework is to lead the observer to gain multiple different sustainability understandings for a system by referring to multiple contexts. This is where the framework significantly deviates from the usual indicator approach (Bossel 1999). This role helps to map sustainability contexts to sustainability boundaries in several ways. One is by indicating different sustainability conditions relevant to different ‘focus–system’ and ‘background’ relationships. Another way is by highlighting the temporal influence of one sustainability or unsustainability condition over others. Such observations allow us to see multiple sustainability boundaries and their changes across time. In addition, by comparing different systems relative to different dimensions and variable conditions that they aggregate, it is conceptually possible to recognize the interlinkage of these boundaries.
(ii) *Uses a complex dynamic observation process supported by reflexive and iterative understanding.* The observation process supported by the framework can be viewed as leading to an emergent process of understanding of a complex phenomenon, which is, in this case, the sustainability of a complex human–natural system. As described earlier, we consider that the basis of sustainability understanding is mainly formed by the interplay of the two dimensions of sustainability-linked knowledge and sustainability-linked worldview (Figure 4a). On this basis, the other dimensions are employed to strengthen the understanding of sustainability further by gaining new observation contexts. In this process, the new reference dimension becomes the cumulative worldview and knowledge, and the observing dimension becomes, e.g., the resource limitation or availability. With the understanding gained through employing these dimensions together, the first sustainability boundary is obtained, whereas in this example, the boundary could be a threshold amount of resources (Figure 4b). Once these limitations are recognized, taking the previously considered dimensions collectively (worldview, knowledge, and resources) as the reference, and then observing possible governing practices to overcome these limitations, a new sustainability boundary can be obtained (Figure 4c). In this process, the previous understanding continues to inform the new understanding. Therefore, the pattern consists of *iterative steps*, where the meaning of iteration in this instance would denote building upon previous steps in understanding.It is also noteworthy that these consecutive understandings need not always have iterative relationships; they can also be independent. For instance, when the resource limitation indicates one sustainability boundary, instead of providing regulation-focused solutions, another possibility is to innovate or create artifacts that can expand the efficiency of resource usage (Figure 4c, d). Referring to contexts that represent alternative preferences (e.g., regulation vs. creativity) would lead to a reflexive decision-making process.
(iii) *Enable to surface complex dynamic sustainability changes*

We noted that changes in sustainability or unsustainability in human–natural systems would be best interpreted as complex dynamic changes. In practice, however, we often tend to focus on one dimension and one particular subsystem. For this reason the conventional problem definitions, solution trajectories, and governance tools could take the form of crisis-response models, where optimizing for a few narrow targets could result in large and unpredicted feedback that may ultimately compromise the resilience of a wider system (Berkes et al. 2003). Failure to recognize multiple contexts and their mutual dependency-induced changing patterns from the outset can easily lead to system collapses. In other words, a response with only one ‘system and background’ unit would generate harmful outcomes in the long run. At the same time, one subsystem may become more significant than other subsystems to interpret a human–natural system’s sustainability or unsustainability at a particular instant. Similarly, one or several dimensions may have heavier significance than others. Some of these ‘system and background’ combinations may be able to trigger system collapses (through theoretically extreme and chaotic situations where the system’s structure collapses) or novel emergent conditions (theoretically making the system entities self-organize to rapidly reach a new stable system structure), by making one combination more critical than others at that particular instant. Such phenomena are visible in processes as creative destruction and reorganization of entity relationships, where rapid change occurs in a relatively short period of time to give rise to fundamentally different system structures and functions.^r^ Otherwise, some of these combinations would maintain the system in a particular stable state by reinforcing the conditions that keep the system in the same state (Figure 3). In this way, the framework facilitates the foresight of a system’s nonlinear sustainability changes by consciously looking for these changes.

## An illustration of the framework

We describe an example based on events that followed on from the discovery of stratospheric ozone depletion to the enactment of international legislations to remedy the issue. Our example aims to show the way to utilize the framework to obtain an evaluation-based holistic sustainability understanding of a system that goes through an unsustainability issue.

Ozone depletion is known as one of the key globally significant, complex dynamic unsustainability issues. It is also significant as paving the path to international environmental policies and laws. In the early 1970s, scientists first observed the damage to the protective ozone layer by man-made atmospheric pollutants. In 1974, they predicted that chlorofluorocarbons (CFCs)—a widely used substance in supersonic jet fuels, aerosol spray cans, and refrigerants—could be the main cause of the damage. In 1985, almost a decade after these predictions, scientists produced direct evidence that ozone depletion was actually occurring, and that the rate of depletion in the ozone over Antarctica was high. In 1987, the Montreal Protocol—the world’s first international environmental convention—was created to set limits on the use of CFCs. Following further research in 1990, measures were taken to strengthen the Montreal Protocol by introducing phase-out commitments for ozone-depleting substances. This included not only CFCs, but also halons and other ozone-destroying chlorine compounds (Levi et al. 1997; Morrisette 1989; Montzka et al. 2011; Andersen and Sarma 2012). With the enacted policies of the Montreal Protocol, the target complete phase-out year for Ozone-depleting substances (ODSs)^s^ was 2005 and most ODSs were to be phased out by 2000. In spring 2006, the ozone hole over Antarctica was arguably the largest recorded. In about the mid 21st century, a notable decrease in the size of the ozone hole is expected to be observed (Newman et al. 2006; WMO 2007; Fahey and Hegglin 2011).^t^

In the process of addressing ozone depletion as a globally as well as locally significant issue, several concerns have competed in the discussion arena for a long time. The perceived environmental and health risk,^u^ the perceived economic impact, and the uncertainty of the issue’s causes and its extent were some of the prominent concerns. Even though it was known by the mid-1970s that CFCs were accumulating in the atmosphere, CFC industry stakeholders and scientists—both in global and national arenas—were skeptical of the need for urgent responses. Because most of the predictions were at a hypothetical stage and were supported only by laboratory model results, many argued that direct evidence of the ozone depletion and the relative magnitude of CFCs as a source of stratospheric chlorine were not yet adequate for concrete actions. CFC manufacturers and customers have argued for delay in regulatory responses until the scientific research could answer these outstanding questions, even though health and environmental organizations continued to insist on rapid actions (Morrisette 1989; Taddonio et al. 2012).^v^ Therefore, this issue was observed for a considerable amount of time before the policies to address the problem became effective. Furthermore, the policy initiatives were to be made by global environmental organizations, which did not have the capacity to enforce direct regulation in individual countries. The stakeholder network around this issue had also been complex with nodes connecting global, national, and institutional levels, which means there were complex feedback and time lags between knowledge generation, worldview changes, policy agreements, and the policies’ actual implementation.

By using the framework, we can attempt to reevaluate the problem so that we recognize the complex dynamic relationships that have played a part in the process of solving it.

The focus–system and the ‘background’ layers could be selected as follows.

Focus–system: A country (that includes the subsystems of economy, society, and eco-system where the issue is experienced)

‘Background’ layer 1: Economic growth or development (that highlights the subsystems of economy and society)

‘Background’ layer 2: Health and ecological conditions depletion (that highlights the subsystems of society and eco-system)^w^

The two layers provide two significant backgrounds with which the focus–system would internalize the issue. To reach an adequate interpretation of sustainability or unsustainability conditions and their changes over time, the mentioned dimensions and, if necessary, some other dimensions could be employed. In our interpretation, we use the same dimensions as those that appear in the description of the framework.

**Figure 5:**
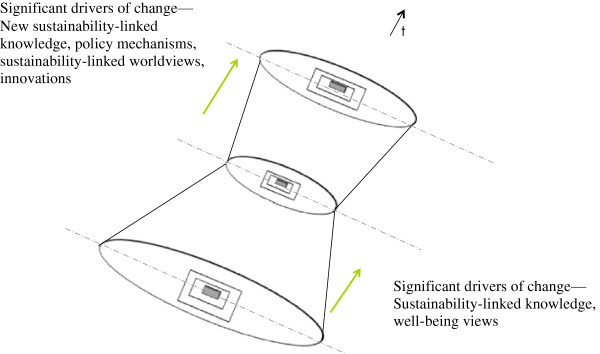
**Dimensions can drive changes in sustainability boundaries of two consecutive states of a ‘system and background’ unit.**

Tables 1 and 2 summarize the sustainability contexts and sustainability boundaries in the form of a matrix. The ‘background’ layers and dimensions together indicate different complex dynamic sustainability contexts (Table 1). Different sustainability boundaries could be obtained by referring to those contexts (Table 2). The boundaries reflect the diverse possible evaluations of sustainability. They could be mapped using actual measurements through indicators, indices, and so on. However, some contexts would not lead to distinctive boundaries, but rather would act as drivers to change the boundaries directly or indirectly by mobilizing feedback processes. For instance, knowledge and technology transfers that were predicted through new governing practices that involve networking and collaborations did not play a role in deciding a specific boundary at a specific state, but being closely attached to the dimensions of ‘sustainability-related knowledge’ and ‘policies, rules, regulations, and governing practices’, have created feedback mechanisms that cumulatively influence other dimensions such as ‘sustainability-linked worldviews’; therefore, they continue to influence the sustainability boundaries of the system in the long run. Once the contexts and boundaries are observed for several states across time, the cumulative change in sustainability boundaries can be visualized as shown in Figure 5.

**Table 1:** **A matrix showing complex dynamic sustainability contexts** ^***a***^ **related to stratospheric ozone depletion issue**

'Background' Layers		Sustainability-linked Knowledge ^***b***^	Sustainability-linked Worldview ^***c***^	Resource limitation/availability	Well-being views	Policies, rules, regulations and governing practices	New creations, innovations and artifacts	'Sustainability-linked Knowledge' + 'Policies, rules, regulations and governing practices' ^***d***^	'Sustainability-linked Knowledge' + 'Sustainability-liked worldviews' + 'New creations, innovations and artifacts' ^***e***^
**Economic growth/development**		Sustainability/unsustainability understanding derived from, (i) knowledge of long-term impact on growth/development of the country related to costs of national health treatments and cost of eco-system restorations (ii) knowledge of impact on growth/development in the process of adopting alternative substances and related technologies^*f*^ (iii) knowledge of different types of stake-holders targeted for ODSs reduction (e.g., in developing countries the stakeholders vary as, importers of products or components where ODSs used, users of ODSs in other manufacturing, producers and users of ODSs^*g*^ etc.)	Sustainability viewed as, (i) the continuous economic growth and development without setbacks (especially from industries' point of view) (ii) positive international trade (and geo-political) partnerships; Sustainablity-views influenced by predominant economic (and related legal and political) views (e.g., those that emphasize the legal rights of citizens [both as global and local citizens] and manufacturers).	Unsustainability issue identified as, (i) the limitation of affordable substitutes to ODSs (CFC-123, CFC-124, HCFCs in early stages and Hydrofluorocarbons [HFCs], Perfluorocarbons [PFCs] and Sulfurhexafluorides [SF_6_] etc later on)^*h*^ (ii) the limitation in applicability of the potential substitutes (e.g., application of compounds in refrigeration, air conditioning, aerosol applications, fire suppression, foam blowing, sterilants, and solvents) that create additional cost of replacement in appliances (iii) the limitation of data and knowledge^*i*^ of cost-effective ODS-substitutes (iv) the limitations in technologies^*j*^ to produce cost-effective ODS-substitutes (v) the limitation of domestic technologies, networks etc to absorb the economic benefits of trade partnerships.	Unsustainability issues identified as disruption of well-being, where well-being is viewed as, (i) the ability to maintain desirable (material) standards of living (that may involve high ODSs emission, such as that of supersonic transport) (ii) the continuous improvement in the living standards (e.g., continuously reducing economic risks related to replacement of ODSs, health research and treatments)^*k*^ (iii) the ability to satisfy same functions, and use same facilities with minimum change to consumption patterns (that involve ODSs and the services and industries that use ODSs such as aerosols, refrigerants etc) to reduce economic impact (iv) the ability to maintain flexibility and adaptability in economic decisions and activities.	Solutions with policies and laws related to, (i) agreements, adaptation schemes and change mechanisms for new substances (e.g., first international discussions under United Nations Environment Programme [UNEP] and World Meteorological Organization [WMO] that led to ‘International Plan of Action’ in 1977; agreements in Vienna convention [1985] by major CFC producers to regulate the compound; commitments with Montreal protocol [1987] to ban the import of ODSs and the discouragement of technologies used for ODSs manufacturing for nonparties (ii) establishing Multilateral Fund [1990] for the implementation of the Montreal Protocol, especially to assist developing countries^*l*^ during the transition process) (iii) the establishment of research networks for global, regional, national and sector-level socio-economic data accumulation.^*m*^ (iv) each country's domestic adjustments to encourage major ODSs producers and small-enterprises for the shift through effective trade mechanisms.^*n*^	Solutions supported by, (i) new evaluator models (e.g., Chemistry-Climate Models [CCM] and related General Circulation Models [GCM]^*o*^) (ii) new technologies that offset additional costs of alternative substances to ODSs (e.g. low cost methods of producing HFC as a refrigerant) (iii) innovative technology transfer mechanisms for adaptation of new substances (e.g., government and industrial partnerships that gave confidence to other manufacturers and part-suppliers to invest on the transition process; industrial leadership pledges for developing countries^*p*^) (iv) innovative market mechanisms to encourage the shift to substitutes, and to eliminate black markets around ODSs and ODS technologies disposal (e.g., the establishment of the grace period, where developing countries could voluntarily reduce ODSs).	Solutions supported by, (i) reevaluating and revising the protocol based on new scientific data and market information [e.g., London Amendments of 1990, the Copenhagen Amendments of 1992, and the Montreal Adjustments of 1997 and 2007, with accelerated phase-out targets, new ODSs and supportive implementation mechanisms] (ii) adopting mechanisms such as trade permits, new global reclaim and recycle mechanisms to reduce the cost of transition while ensuring proper destruction of ODSs.	Sustainability achieved through, (i) continuation to look for innovative solutions supported by long-term investments (e.g., research on Geo-Engineering Solutions as solar radiation-management [SRM], where SRM aims to reduce solar wave radiation before it reaches earth via methods such as injecting aerosols to atmosphere to reflect sunlight (ii) continuation of international partnerships to generate cost-effective alternative solutions (e.g., produce and use more ozone friendly as well as energy efficient technologies and appliances that have added benefits to both producers and consumers).^*q*^
**Health and ecological conditions depletion**		Sustainability/unsustainability understanding derived from, (i) knowledge of environmental related health impact of the issue (e.g., ecological imbalance, cancer by UV-B) (ii) knowledge of ODSs (e.g., chemistry of substances and reactions) (iii) knowledge of ozone depletion chemistry, stratosphere conditions and cycle patterns (e.g., polar stratospheric clouds [PSCs], dynamical structure of polar winter and spring stratosphere, stratosphere and troposphere coupling) (iv) knowledge of alternative substances and technologies with their added environmental benefits (v) nonknowledge and nesciences on future conditions (e.g., future discoveries such as, the additional UV-B impacts on human health, ODS-substitutes' impact on phenomena such as global warming, the health impact distribution among countries, changes to the expected trends due to unanticipated causes^*r*^ etc).	Sustainability viewed in terms of importance/non-importance of, (i) sustained human health (ii) eco-system well-being, and the perceived degree of autonomy and responsibility for them; Sustainability-linked views supported by world-centric and group- (nations, locality) centric ideas on the environmental (and related health) impact, and by the related sense of responsibility; Sustainability-liked views that influence the extent of comfort with health and ecological risks (risk in the face of dread, familiarity and extent of exposure).^*s*^	Unsustainability issues identified as, (i) the limitation of known substitutes for ODSs^*t*^ (ii) the limitations in applicability of new substitutes in existing appliances and technologies (iii) the limitations in available technologies to reduce ODS emissions (e.g., difficulties faced in producing non-harmful CFC varieties by CFC manufacturers) (iv) the limitations in available scientific data to verify the extent of the health and ecological impact (e.g., the lack of evidence of ozone depletion and specific causes, and other scientific uncertainties related to ODS-substitutes).	Unsustainability issues identified as disruption of well-being, where well-being is viewed as, (i) the ability to maintain desirable (health and eco-system related) living standards (ii) positive conditions to support good human health and eco-system balance (e.g., reduced level of UV-B radiation through reduced ozone depletion rate) (iii) the continuous improvement of the (health and eco-system related) living standards (e.g., continuous reduction of cancer risks and negative effects on aquatic biochemical cycles^*u*^).	Solution with policies and laws related to, (i) ODSs emission reduction and complete elimination mechanisms (supported mainly by the Montreal Protocol) (ii) global, regional, national research network establishment for new health and ecological related data accumulation (e.g., commitments for assessments of national limits set for ODSs production and consumption in every four years; national policies that support research on UV-B effect on health, and on terrestrial and aquatic eco-systems; international policy initiatives such as World Plan of Action for the Ozone Layer [1977] by United Nations Environment Program [UNEP]).	Solutions supported by, (i) technologies to produce and utilize alternative substances with improved health and ecological benefits (e.g., producing HCFC as an alternative for CFC (especially producing HCFC-225 between 1990 and 1994, to replace CFC-113)^*v*^ (ii) efficient technology transfer mechanisms for efficient adaptation of new substances (e.g., partnerships between government supported environmental agencies [e.g., Environmental Protection Agency-EPA] and major industries in assessing and adapting new technologies, that accelerated the substitution process) (iii) innovative trade, policy mechanisms to encourage the shift to substitutes and further research.	Solutions supported by reevaluating and revising the protocol based on new scientific information (e.g., London Amendments of 1990, the Copenhagen Amendments of 1992, and the Montreal Adjustments of 1997 and 2007 that introduced new ODSs and accelerated the phase-out targets, such as the accelerated phase-out plan for HCFCs and methylbromide considering their underestimated rate of threat to ozone layer and their contribution to global-warming as a green house gas; supportive assessments made in 1989, 1991 and 1994 with panels representing science, economy and technology).	Sustainability achieved through, (i) continuously look for innovative solutions to reduce health and ecological impact (e.g., research on Geo-Engineering Solutions as solar radiation-management [SRM], where SRM aims to reduce solar wave radiation before it reaches earth via methods such as injecting aerosols to atmosphere to reflect sunlight (ii) continuation of international partnerships to reduce ODSs (iii) improvements of recycle and reclaiming technologies and mechanisms (iv) treating environmental issues not as isolated issues, but interrelated issues of global human–natural system (e.g., produce and use more ozone friendly as well as energy efficient technologies and appliances).^*w*^

^*a*^These contexts as described elsewhere, are observation metastructures in the evaluation process.

^*b,c*^To maintain presentation simplicity, only the key dimensions are shown as the column titles. However, it is important to note that in addition to the shown explicit roles, the two dimensions of sustainability-linked knowledge and sustainability-linked worldview also play background roles to other dimensions in the process of defining sustainability contexts.

^*d,e*^Similarly, other dimension combinations also would enable us to see more contexts by supporting a reflexive and iterative understanding process; we show only two significant examples.

^*f*^E.g., new compounds and related technologies in refrigeration, air conditioning, aerosol applications, fire suppression, foam blowing, sterilants, and solvents.

^*g*^As specified by Munasinghe and King (1991); adopted from Taddonio et al. (2012).

^*h*^In the early stage, the chemical industry was working to produce new chemicals such as CFC-123, and CFC-134; however, these developments were controlled by the chemistry and the market (Morrisette 1989). The limitations of the available ODS-substitutes made them essential resources in this issue.

^*i,j*^Just as ODS-substitutes, the related knowledge, and the technologies to produce them also are considered as resources.

^*k*^Beyond the distinctive catastrophic nature, the health risks also generate long-term economic impacts for a country.

^*l*^Developing countries that consume less than 0.3 kilograms of ODSs per person per year are known as ‘Article 5 countries’.

^*m*^E.g., International Council of Scientific Union (ICSU), United Nations Environment Programme (UNEP), the World Meteorological Organization (WMO).

^*n*^E.g., The domestic policies adopted by the European Commission (EC) to allow the use of HCFC as a solvent and foam production supplement, but ban for the use in some types of refrigeration and air-conditioning services; and later to ban all use and imports of products that use HCFC. This stepwise approach is believed to have encouraged the small and medium scale companies to be more innovative in developing alternatives, and to transfer to HCFC-free technologies (Taddonio et al. 2012). Another such mechanism is the tradable permits that were adopted by many countries, which aimed for flexibility during the transition process, while at the same time ensuring the phase-out schedules are met and the ODSs are destroyed effectively.

^*o*^Of World Meteorological Organization (WMO) and United Nations Environment Program (UNEP) (Eyring et al. 2005; Perlwitz et al. 2008).

^*p*^E.g., Pledge by automotive community to (i) recycle (ii) phase-out CFC-12 (in 1988 and 1990); Voluntary phase-out of CFC foam in food packaging; Pledge by Japanese enterprises to phase-out ODSs use at their facilities in developing countries within one year of the phase-out at domestic facilities (in 1990) (Taddonio et al. 2012).

^*q,w*^Such change in direction of the nature of envisioned solutions is heavily influenced by changed worldviews, which may have influenced by factors such as, increased acceptance of irreversibility of harm already occurred, acceptance of the close connection of ozone depletion issue and global warming issue (such as the man-made nature, and the possible dynamic interrelation [Andersen and Sarma 2012]), the increased trust towards the functionality of global protection initiatives, the increased dependency upon technology based solutions, and so on. Further, they require views that support nonknowledge-based actions, which may have become more acceptable with time.

^*r*^Such as the effect on ozone concentration by stratospheric sulfate particles from volcanic eruptions (e.g. Mt Pinatubo eruption in 1991), varying temperature in stratosphere (due to winter time polar vortex circulation and solar cycle variations), atmospheric dynamics (which is heavily influenced by increased carbon dioxide emissions), abundance of trace gases such as water vapor, methane and N_2_O (atmospheric N_2_O has increased in recent times due to high fertilizer use), and so on (Weatherhead and Andersen 2006). These dynamic factors would continue to exert uncertainty for the rate of recovery of ozone and its future stabilizing concentration.

^*s*^Factors that affect the risk perception as categorized by Slovic (1987); adopted from Morrisette (1989).

^*t*^With limited scientific knowledge of exact cause of the ozone layer depletion, only few substitutes were identified in the beginning.

^u^The disruption to aquatic biochemical cycle is found to reduce the production of phytoplankton, and to lower the reproductive capacity of aquatic life such as fish, shrimp and crabs (Worrest and Häder 1989).

^*v*^Resistance from key producers to halt CFCs without adjustable alternatives had been one of the key bottlenecks in implementing the Montreal Protocol. Finding alternatives for CFCs—especially for the widely used ones—is believed to have accelerated the process (Taddonio et al. 2012).

**Table 2:** **Possible sustainability boundaries related to the identified sustainability contexts related to stratospheric ozone depletion issue**

'Background' Layers		Sustainability-linked Knowledge	Sustainability-linked Worldview	Resource limitation/availability	Well-being views	Policies, rules, regulations and governing practices	New creations, innovations and artifacts	'Sustainability-linked Knowledge' + 'Policies, rules, regulations and governing practices'	'Sustainability-linked Knowledge' + 'Sustainability-liked worldviews' + 'New creations, innovations and artifacts'
**Economic growth/development**		Boundary: Maximum acceptable cost values related to replacements of ODSs; Minimum accepted negative change in values of growth rate, per capita income, gross national production etc, related to ODS-replacement and long-term health impact costs; Thresholds that represent the predictive capacity of development and growth rate changes.	Boundary: Values of growth and development related sustainability indices (especially with respect to values that reflect public perception of investments in environmental issues, degree of responsibility, and associated economic risk).	Boundary: Minimum accepted change in values of growth rate, per capita income, gross national production and related sustainability indices that reflect the costs of ODSs-replacement process with available substitutes and appliances; Related sustainability index values.	Boundary: Accepted same-lifestyle based well-being index (and related sustainability index) values (that consider the economic impact of ODS non-replacing scenario, such as the increased long-term costs on health research and treatment); Accepted alternative lifestyle based index values (that consider the long-term economic impact of the replacement of ODSs, general perception of economic risks, impact of trade partnerships etc).	Boundary: Minimum achievable (and acceptable) development and growth values predicted for the optimal function of, regulations and mechanisms in Montreal Protocol, supporting trade policies, and domestic reduction policies; Sustainability index values that take in to account the expected impact of the policy mechanisms.	Boundary: Values from new sustainability evaluation models (that incorporate new cost indicators, long-term growth and development indicators that consider the impact from adoption of new technologies, ODS-substitutes, new recovery rates, new market mechanisms, and new technology transfer mechanisms).	Boundary: Sustainability index values that consider the maximum accepted negative changes in cost, growth, and well-being indicators from new reduction and phase-out targets (with newly recognized ODSs, and improved substitution-, trade-, and disposal- mechanisms).	Boundary: Sustainability index values measured considering the economic impact of new phase-out methods supported by emerging technology-based solutions, improved trade mechanisms, holistic scientific models, and the changed perception of sustainable solutions.
**Health and ecological conditions depletion**		Boundary: Minimum recoverable ozone level with ODS-substitutes; Threshold of available verifiable scientific data related to issue; Minimum knowledge to predict possible catastrophic conditions (e.g., discovery of ozone hole over Antarctica, proof of cancer risk); Boundaries of nonknowledge and nesciences related to the issue; Boundaries of knowledge specified in other cells.	Boundary: Values of public perception based sustainability indices that reflect the projected impact on health and eco-system, the degree of responsibility for environmental issues, and the level of associated health and ecological risk.	Boundary: Minimum recoverable ozone level with available ODS-substitutes and related alternative appliances; Stratospheric ozone layer recovery rate; Threshold of available scientific data related to the issue; Already available technology level to ensure the ODSs replacement; Related sustainability index values.	Boundary: Alternative/same-lifestyle based well-being index (and related sustainability index) values that consider the measures of health and ecological depletion/improvement (e.g., stratospheric ozone layer recovery rate, rate of reduction/increase of ozone hole size over the Antarctica, ODS level in stratosphere, current emission reduction and freezing capacity of ODSs, and reduced/increased cancer risk).	Boundary: Minimum achievable environment protection/replenishment targets predicted by the optimal function of, regulations and mechanisms in the Montreal Protocol, supporting trade policies, and domestic reduction policies; Sustainability index values that take in to account the expected impact of the policy mechanisms.	Boundary: Values from new sustainability evaluation models (that incorporate the impact on health and eco-system-sustainability by considering new technologies, ODS-substitutes, new recovery rates, new market mechanisms, new technology transfer mechanisms, and related ozone recovery rate).	Boundary: Sustainability index values that reflect health and eco-system well-being improvements from new reduction and phase-out targets (with newly recognized ODSs, improved substitution-, trade-, and disposal-mechanisms, and new values of the expected ozone recovery rate).	Boundary: Sustainability index values that reflect health and eco-system well-being improvements (measured considering new phase-out targets supported by emerging technology-based solutions, improved trade mechanisms, holistic scientific models, and the changed perception of sustainable solutions).

The discovery of ozone depletion significantly shrank the sustainability boundaries in general from their previously perceived limits. In other words, the sustainable operating space of the planet Earth, and therefore of any particular country, was decreased. This trend continued with subsequent verifications based on new, yet incomplete knowledge^x^. Furthermore, it had taken a longer time to discover the ODSs, and to identify alternative substances that could offset the impact, and a greater number of years to produce sound policy and operational mechanisms that connect global, regional, national, and institutional levels. This situation is reflected by the change in boundary in the bottom half of the sustainability sphere (Figure 5). The middle cross-section shows a situation where positive (sustainability) drivers and conditions have overtaken the negative (unsustainability) drivers and conditions. To make such an evaluation, not only the current conditions and feedback or feedforward effects, but also the anticipated future conditions and effects are necessary. To simplify the process, we would consider the transition state to be the state where all necessary policy enactments were made within the country to halt or reduce emissions. The expanding boundaries in the sustainability sphere’s upper section suggest the continuation of the same pattern. One significant feedforward in this expanding stage could be the changing views and perceptions of the human–system towards the global environment accompanied by a sense of responsibility towards global well-being. However, as past actions have already created a certain degree of irreversible harm to the sustainability boundaries, conceptually they need to be visualized in a way that the sustainability operating space remains smaller than it was before the issue was discovered.

Likewise, using the framework we can evaluate the sustainability or unsustainability of a human–natural system. The contexts and boundaries provide evaluation-based understanding of the ‘whole’, by referring to ‘parts’ of a complex dynamic phenomenon. One significant limitation in this example is that it is based on an already well-known and well-documented issue whose complexities are already dealt with. Therefore, complex dynamics could be observed only in retrospect. However, using the framework to analyze a system that experiences an unsolved ongoing issue (or several interlinked issues) across multiple time-spans, would more actively facilitate the observer’s own understanding process as described earlier. Another factor to note is that with the current example we have tried to generally demonstrate the way the focus–system, ‘background’ layers, and dimensions can be selected and how they could be used in sustainability evaluation. However, if we selected a specific country or a similar system, for an in-depth case study, local specificities could also be integrated to identify distinctive patterns of change.

## Conclusion

The proposed framework was developed to incorporate some of the basic ideas of complex dynamics linked to the sustainability of human–natural systems, and the complex dynamics linked to observing sustainability in these systems. First, we propose the concept of ‘sustainability boundaries,’ with which different sustainability contexts are mapped to an evaluation-based understanding of sustainability. Two complementary methods are proposed to observe sustainability boundaries. By introducing a ‘system and background’ unit as an observation unit, the layer view-based method explores ways of recognizing multiple sustainability boundaries in a relatively fixed time frame. The mechanisms involved in these observations are grounded in some of the key complexity ideas. The observation process supported by this method is argued to be a complex dynamic process in itself. Second, a process that enables us to observe sustainability boundaries under variable sustainable or unsustainable conditions is introduced. The ‘system and background’ units are examined further to separately consider system relationships and other explicit sustainability or unsustainability conditions. Sustainability or unsustainability conditions are observed through a set of dimensions. Brief descriptions for each selected dimension and their roles in forming and changing sustainability boundaries are provided. By combining these two complementary methods into an observation process, we argue that observing human–natural systems relative to layers and dimensions enable the production of multiple different sustainability contexts. The observation process represents an integrated differentiating, analysis, and synthesis process that translates sustainability contexts to conceptual sustainability boundaries (visualized as a sustainability sphere). The framework allows us to address sustainability contexts systemically by engaging the researcher in a reflexive and iterative understanding process. Reflexive and iterative understanding are two of the key mechanisms involved in observing the complex dynamics in a stringent manner. In this way, the framework would increase the observer’s capacity to reflect the complex dynamics consciously, therefore, may have significant implications for real-world sustainability appraisal.

## Endnotes

^a^Over the years, the objective significance of the concept of ‘sustainable development’ has been interpreted and enriched with diverse research perspectives that also have led to a slightly different concept of ‘sustainability,’ which can be regarded as encompassing the subjective and normative characteristics of the original concept with a wider scope. A rich description for how this concept has evolved over the years can be found in the historical and conceptual reviews by Kidd (1992) and Mebratu (1998).

^b^From the simplest perspective, complex dynamics are viewed as patterns in systems that result from the system agents or objects and the interactions among them (derived from definitions by Maturana and Varela (1987), Miller and Page (2009), Juarrero (2002), and Morin (2008)).

^c^By organizing relationships, we mean the feedback- and emergent properties-based complex relationships such as self-organizing.

^d^There are many interpretations of a ‘system’ (Bailey 1994). In this instance, following interpretations by Meadows (1999, 2008), a system is regarded as a group of entities that are connected by common behavior pattern(s).

^e^In these studies, human–natural systems were regarded as coupled systems where complex evolutional adaptations are brought into focus.

^f^Other related concepts such as beliefs and mindsets are also included within the term, ‘views’.

^g^Here the term ‘cognitive distance’ is not used in a strict sense; however, the use of the term here does not contradict with how it is used in the field of psychology. In psychology, cognitive distance refers to people’s beliefs about distances between places in large-scale spaces, places that are far apart and obscured as not to be visible from each other, while in contrast, perceptual distance refers to people’s beliefs about distances between places that are visible from each other (Montello 1991). For the distance involved in focus and background layers, both the information explicitly perceived and information not explicitly perceived are involved; therefore, in this instance, ‘cognitive distance’ is regarded as being more appropriate.

^h^More specifically, primary understanding can be gained by interpreting sustainability in relation to the focus–system. Subsidiary understanding represents sustainability understanding that is obtained by referring to its relationship with background (Polanyi 1974; Polanyi and Prosch 1977). Furthermore, by the term ‘holistic,’ we aim to represent the understanding that encompasses the understanding of both parts and the whole.

^i^Salas-Zapata et al. (2012) highlight that the lack of a set of principles for knowledge construction is one of the most prominent issues hindering the advance of sustainability science. However, it is also important to be cautious in using the term ‘sustainability principles.’ There are multiple set of principles developed by the sustainability discourse, which often reflect the parent disciplines and focuses that range across conceptual (Dovers and Handmer 1992; Turner et al. 2003; Dresner 2008), systemic, ecological (e.g., deep ecological principles), economic (e.g., principles of triple bottom line), policy, and operational (e.g., United Nations Global Compact, operation principles of sustainability [Daly 1990]) perspectives. Rather than sticking to any specific domain, here we focus on some of the general sustainability principles that we regard as being especially relevant in indicating and influencing sustainability conditions in a complex dynamic context. These principles vary from one system to another; therefore, they may include both the general overarching principles and the context-specific principles.

^j^Explicit knowledge is formalized and codified, and is sometimes referred to as ‘know-what’ (Brown and Duguid 1998). Therefore, it is more straightforward to identify, store, and retrieve (Wellman et al. 1992). Tacit knowledge was originally defined by Polanyi (1966) and is sometimes referred to as know-how. It refers to intuitive, hard-to-define knowledge that is largely based on experience. Because of this, tacit knowledge is often context dependent and personal in nature. It is hard to communicate and deeply rooted in action, commitment, and involvement (Horvath 2000; Nonaka 2002; Collins 2010). Embedded knowledge refers to the knowledge that is locked in processes, products, culture, routines, artifacts, or structures (Horvath 2000; Gamble and Blackwell 2001). This knowledge is embedded either formally, such as through a management initiative to formalize a certain beneficial routine, or informally when the organization uses and applies the other two types of knowledge.

^k^By agents, we mean individuals and cohesive groups such as networks and societies.

^l^While metastructure is a term found in studies of ontology, Beckers (2012) introduces the concept of metastructures to analyze these clusters in detail. He defines a metastructure as a historically evolved structure composed of four elements—(i) basic assumptions, (ii) basic evaluations, (iii) driving forces, and (iv) institutionalizations—that substantially affect societal and individual thoughts, actions, and relationships. Beckers (2012) explores the implications of metastructures in the formation of an ethical understanding of sustainability. A metastructure related to observation can be further identified as a system of thoughts (Jenks 2004).

^m^These stable views are similar to the layers of understanding indicated in spiral dynamics and also to the hierarchical layers found in cognitive and personal development (Maslow et al. 1970; Beck and Cowan 1996); however, the mechanisms involved are different, especially because the notion of hierarchy is not strictly included in the interpretation.

^n^Complex thinking could be understood as a next step in the line of systems thinking and holistic thinking. As indicated earlier, the significance is that, with complex thinking, the system relationships are seen in a more complex manner, rather than leading to a simplified or generalized understanding. By being aware of multiple boundaries and treating them both separately and collectively, complex thinking would lead to holistic understanding.

^o^More precisely, under his view of complex thinking, Morin (2008) formulated seven interrelated and complementary principles. These are the organizational and system principle, the principle of the hologram, the principle of feedback, the principle of the recursive loop, the principle of autonomy/dependence (self-eco-organization), the principle of dialogue, and the principle of reintroduction of a cognitive subject in the cognitive processes (see Morin 2008, pp. 112).

^p^Including the significant issues that highlight those relationships.

^q^According to terminology and definitions by systems scientists (see Miller (1978), adopted from Bailey (1994)).

^r^A detailed explanation can be found for the mechanisms of creative destruction processes in relation to complex adaptive systems in Holling (1986) and Berkes et al. (2003).

^s^Widely used ODSs are CFCs, halons, carbon tetrachloride, trichloroethane, hydrobromofluorocarbons and hydrochlorofluorocarbon (HCFC). In the beginning HCFC was used as a substitute for some CFCs, but later was also substituted with other compounds such as hydrofluorocarbon (HFC) that have zero ozone depletion threat.

^t^Along with stratospheric ozone being stabilized at levels observed before 1980.

^u^Stratospheric ozone shields the earth from harmful UV radiation (UV-B). Increase in the UV radiation could cause significant health damage (Lippmann 1989; Norval et al. 2011). The biggest concern was the fear that they directly cause skin cancers.

^v^While these arguments were also because of differences in the interest of stakeholder groups, some nations have showed reluctance to cooperate in international dialogues because of the locally politicized nature of how this issue was discussed in its first stage, especially in the United States, which shows how geopolitical concerns influence the decisions with respect to local economies and environment (Morrisette 1989).

^w^The first ‘background’ layer—‘economic development or growth’, is considered here as representing the economic system and the economy related social system therefore predominantly the human system of a country. The second ‘background’ layer—‘health and ecological conditions depletion’, is considered as representing the eco-system and the social system therefore the combination of a human–natural system. The impacts to the social system considered in the two ‘backgrounds’ differ with respect to urgency, irreplaceability and irreversibility.

^x^Having suggested the existence of the issue, yet being unable to verify exact causes, new knowledge (especially the nonknowledge and nesciences as indicated in Table 1) have strengthened the uncertainty, hence the friction for policy initiatives.
